# Resilience to Alzheimer's disease in individuals with Down syndrome

**DOI:** 10.1002/alz70855_099390

**Published:** 2025-12-23

**Authors:** Lisi Flores Aguilar, Sierra Wright, Jillian V Berry, Jerry Jierui Lou, Jeremy P. Rouanet, Natalie C. Edwards, William H Yong, Mari Perez‐Rosendahl, Dan Hoang, Brianna Gawronski, Lourdes Gonzalez, Kevin Wood, Ann‐Charlotte Granholm, Elliott J. Mufson, Edwin Monuki, Milos D. Ikonomovic, Julia K. Kofler, Eric Doran, Frederick A Schmitt, Jordan P. Harp, Jose Gutierrez, Patrick J. Lao, Donna M. Wilcock, Adam Brickman, Elizabeth Head, Alzheimer's Biomarker Consortium — Down Syndrome (ABC‐DS)

**Affiliations:** ^1^ University of California, Irvine, Irvine, CA, USA; ^2^ University of California Irvine, Irvine, CA, USA; ^3^ Columbia University, New York, NY, USA; ^4^ University of Colorado, Aurora, CO, USA; ^5^ Barrow Neurological Institute, Phoenix, AZ, USA; ^6^ University of Pittsburgh, Pittsburgh, PA, USA; ^7^ University of Kentucky, Lexington, KY, USA; ^8^ Indiana University School of Medicine, Department of Neurology, Indianapolis, IN, USA

## Abstract

**Background:**

Individuals with Down syndrome (DS) are at increased genetic risk of developing Alzheimer's disease (AD). By the age of 40 years, most individuals with DS exhibit AD neuropathology, and dementia in their early 50s. Similar to other genetic forms of AD, despite having a high burden of AD neuropathology, some individuals with DS do not develop dementia. Our objective was to investigate the proportion of individuals resilient to AD in a clinically and neuropathologically characterized autopsy cohort of individuals with DS.

**Methods:**

This is a cross‐sectional autopsy cohort study of individuals with DS from the University of California, Irvine ADRC who had available neuropathology data, including measures of AD neuropathology and other co‐pathologies (*n* = 67, 37‐60 years old, 60% women). Dementia status was assigned via an expert consensus panel by review of the cognitive tests, questionnaires, health history and clinical exam data. For this study, individuals with DS resilient to AD were defined as those without dementia but at a similar age and level of AD neuropathology than those individuals with DS with dementia.

**Results:**

At their last cognitive evaluation, 56 individuals with DS had AD dementia (43‐66 years old), two had possible AD dementia (45 and 61 years old), and nine did not have dementia (37‐70 years old). For those without dementia, the average time between their last neuropsychological examination and death was 9 ± 4 months. Out of the nine individuals with DS without dementia, 1 was at Braak stage 0 and had no neuritic plaques (37 years old). The other eight cases (42‐70 years old) exhibited intermediate to high AD neuropathological change (Braak stage III‐VI, Thal phase 3‐5). Regarding co‐pathologies, cerebral amyloid angiopathy was the most frequent (55%); TDP‐43 pathology was present in one case, Lewy body pathology in two cases, and none of them exhibited hippocampal sclerosis.

**Conclusion:**

In the UCI ADRC autopsy cohort of individuals with DS, ∼14% of individuals with DS over 40 years old did not have dementia at their last clinical evaluation before death and showed intermediate to high AD neuropathological change suggesting that they may have been resilient to disease.

